# Supplementary material for: Unveiling the Bioactive Potential of the Invasive Jellyfish Phyllorhiza punctata Through Integrative Transcriptomic and Proteomic Analyses
Source: Biomolecules. 2025 Aug 4;15(8):1121. doi: 10.3390/biom15081121 (PMC12383608; doi:10.3390/biom15081121)
Supplement: Supplementary file 1 [file biomolecules-15-01121-s001.zip › Suplementary Material/FileS3/PEPFOLD4/XP_065062503_11164/clusters.html]

 PEP-FOLD clusters


Download archive of all models  here   

|  |  |  |
| --- | --- | --- |
| # | file | sOPEP |
| # cluster 1 - Size: 58 |  |  |
|  | XP\_065062503\_11164-model1 | -15.7896 |
|  | XP\_065062503\_11164-model1.1 | -15.6469 |
|  | XP\_065062503\_11164-model1.2 | -15.5851 |
|  | XP\_065062503\_11164-model1.3 | -15.5469 |
|  | XP\_065062503\_11164-model1.4 | -15.5467 |
|  | XP\_065062503\_11164-model1.5 | -15.528 |
|  | XP\_065062503\_11164-model1.6 | -15.528 |
|  | XP\_065062503\_11164-model1.7 | -15.5261 |
|  | XP\_065062503\_11164-model1.8 | -15.5125 |
|  | XP\_065062503\_11164-model1.9 | -15.5037 |
|  | XP\_065062503\_11164-model1.10 | -15.5037 |
|  | XP\_065062503\_11164-model1.11 | -15.4985 |
|  | XP\_065062503\_11164-model1.12 | -15.4953 |
|  | XP\_065062503\_11164-model1.13 | -15.4873 |
|  | XP\_065062503\_11164-model1.14 | -15.4873 |
|  | XP\_065062503\_11164-model1.15 | -15.4794 |
|  | XP\_065062503\_11164-model1.16 | -15.4728 |
|  | XP\_065062503\_11164-model1.17 | -15.4616 |
|  | XP\_065062503\_11164-model1.18 | -15.4616 |
|  | XP\_065062503\_11164-model1.19 | -15.4603 |
|  | XP\_065062503\_11164-model1.20 | -15.458 |
|  | XP\_065062503\_11164-model1.21 | -15.4499 |
|  | XP\_065062503\_11164-model1.22 | -15.4499 |
|  | XP\_065062503\_11164-model1.23 | -15.4496 |
|  | XP\_065062503\_11164-model1.24 | -15.4356 |
|  | XP\_065062503\_11164-model1.25 | -15.4261 |
|  | XP\_065062503\_11164-model1.26 | -15.4144 |
|  | XP\_065062503\_11164-model1.27 | -15.4029 |
|  | XP\_065062503\_11164-model1.28 | -15.4019 |
|  | XP\_065062503\_11164-model1.29 | -15.3971 |
|  | XP\_065062503\_11164-model1.30 | -15.3749 |
|  | XP\_065062503\_11164-model1.31 | -15.3614 |
|  | XP\_065062503\_11164-model1.32 | -15.3554 |
|  | XP\_065062503\_11164-model1.33 | -15.3531 |
|  | XP\_065062503\_11164-model1.34 | -15.3475 |
|  | XP\_065062503\_11164-model1.35 | -15.3416 |
|  | XP\_065062503\_11164-model1.36 | -15.3389 |
|  | XP\_065062503\_11164-model1.37 | -15.3373 |
|  | XP\_065062503\_11164-model1.38 | -15.3314 |
|  | XP\_065062503\_11164-model1.39 | -15.3277 |
|  | XP\_065062503\_11164-model1.40 | -15.311 |
|  | XP\_065062503\_11164-model1.41 | -15.31 |
|  | XP\_065062503\_11164-model1.42 | -15.3091 |
|  | XP\_065062503\_11164-model1.43 | -15.286 |
|  | XP\_065062503\_11164-model1.44 | -15.2785 |
|  | XP\_065062503\_11164-model1.45 | -15.2606 |
|  | XP\_065062503\_11164-model1.46 | -15.2568 |
|  | XP\_065062503\_11164-model1.47 | -15.2359 |
|  | XP\_065062503\_11164-model1.48 | -15.2354 |
|  | XP\_065062503\_11164-model1.49 | -15.2251 |
|  | XP\_065062503\_11164-model1.50 | -15.2251 |
|  | XP\_065062503\_11164-model1.51 | -15.2147 |
|  | XP\_065062503\_11164-model1.52 | -15.2106 |
|  | XP\_065062503\_11164-model1.53 | -15.209 |
|  | XP\_065062503\_11164-model1.54 | -15.1654 |
|  | XP\_065062503\_11164-model1.55 | -15.1261 |
|  | XP\_065062503\_11164-model1.56 | -15.0887 |
|  | XP\_065062503\_11164-model1.57 | -15.0441 |
| # cluster 2 - Size: 40 |  |  |
|  | XP\_065062503\_11164-model2 | -15.6005 |
|  | XP\_065062503\_11164-model2.1 | -15.5964 |
|  | XP\_065062503\_11164-model2.2 | -15.5958 |
|  | XP\_065062503\_11164-model2.3 | -15.5305 |
|  | XP\_065062503\_11164-model2.4 | -15.5071 |
|  | XP\_065062503\_11164-model2.5 | -15.4891 |
|  | XP\_065062503\_11164-model2.6 | -15.4866 |
|  | XP\_065062503\_11164-model2.7 | -15.4595 |
|  | XP\_065062503\_11164-model2.8 | -15.454 |
|  | XP\_065062503\_11164-model2.9 | -15.4482 |
|  | XP\_065062503\_11164-model2.10 | -15.445 |
|  | XP\_065062503\_11164-model2.11 | -15.4168 |
|  | XP\_065062503\_11164-model2.12 | -15.4162 |
|  | XP\_065062503\_11164-model2.13 | -15.4018 |
|  | XP\_065062503\_11164-model2.14 | -15.3876 |
|  | XP\_065062503\_11164-model2.15 | -15.3824 |
|  | XP\_065062503\_11164-model2.16 | -15.3778 |
|  | XP\_065062503\_11164-model2.17 | -15.376 |
|  | XP\_065062503\_11164-model2.18 | -15.3759 |
|  | XP\_065062503\_11164-model2.19 | -15.3708 |
|  | XP\_065062503\_11164-model2.20 | -15.3664 |
|  | XP\_065062503\_11164-model2.21 | -15.3613 |
|  | XP\_065062503\_11164-model2.22 | -15.3568 |
|  | XP\_065062503\_11164-model2.23 | -15.3415 |
|  | XP\_065062503\_11164-model2.24 | -15.3274 |
|  | XP\_065062503\_11164-model2.25 | -15.3225 |
|  | XP\_065062503\_11164-model2.26 | -15.3062 |
|  | XP\_065062503\_11164-model2.27 | -15.2959 |
|  | XP\_065062503\_11164-model2.28 | -15.2904 |
|  | XP\_065062503\_11164-model2.29 | -15.2865 |
|  | XP\_065062503\_11164-model2.30 | -15.2855 |
|  | XP\_065062503\_11164-model2.31 | -15.2778 |
|  | XP\_065062503\_11164-model2.32 | -15.2464 |
|  | XP\_065062503\_11164-model2.33 | -15.2154 |
|  | XP\_065062503\_11164-model2.34 | -15.2145 |
|  | XP\_065062503\_11164-model2.35 | -15.1938 |
|  | XP\_065062503\_11164-model2.36 | -15.1255 |
|  | XP\_065062503\_11164-model2.37 | -15.0663 |
|  | XP\_065062503\_11164-model2.38 | -15.0316 |
|  | XP\_065062503\_11164-model2.39 | -14.8633 |
| # cluster 3 - Size: 2 |  |  |
|  | XP\_065062503\_11164-model3 | -14.6162 |
|  | XP\_065062503\_11164-model3.1 | -14.4863 |
|  |  |  |
